# PdAg Nanoparticles within Core-Shell Structured Zeolitic Imidazolate Framework as a Dual Catalyst for Formic Acid-based Hydrogen Storage/Production

**DOI:** 10.1038/s41598-019-52133-5

**Published:** 2019-10-30

**Authors:** Meicheng Wen, Kohsuke Mori, Yuya Futamura, Yasutaka Kuwahara, Miriam Navlani-García, Taicheng An, Hiromi Yamashita

**Affiliations:** 1 Division of Materials and Manufacturing Science, Graduate School of Engineering, Osaka University, 2-1 Yamadaoka, Suita, Osaka 565-0871 Japan; 2 JST, PRESTO, 4-1-8 Honcho, Kawaguchi, Saitama 332-0012 Japan; 3 Elements Strategy Initiative for Catalysts Batteries (ESICB), Kyoto University, Katsura, Kyoto 615-8520 Japan; 4 Guangzhou Key Laboratory of Environmental Catalysis and Pollution Control, School of Environmental Science and Engineering Institute of Environmental Health and Pollution control, Guangdong University of Technology, Guangdong, 51006 China

**Keywords:** Heterogeneous catalysis, Metal-organic frameworks

## Abstract

Formic acid (FA; HCOOH) is one of the most promising candidates for the storage of hydrogen (H_2_). Herein, we report a H_2_ storage/production system based on the hydrogenation of CO_2_ and dehydrogenation of FA, using a nanostructured heterogeneous catalyst. Pd_1_Ag_2_ nanoparticles with an average size of 2.8 nm were encapsulated within a zeolitic imidazolate framework (ZIF-8) having a core-shell structure (ZIF-8@Pd_1_Ag_2_@ZIF-8). This composite displayed high activity and stability during both the hydrogenation of CO_2_ to produce FA and the dehydrogenation of FA into H_2_ and CO_2_. This improved performance is attributed to the use of ultrafine Pd_1_Ag_2_ nanoparticles as well as the spatial regulation of the nanoparticles within the reaction field. This study suggests a new strategy for controlling the spatial distribution of metal nanoparticles within MOFs so as to fine-tune the catalytic activity and selectivity of ZIF-8@metal nanoparticles@ZIF-8 catalysts.

## Introduction

Hydrogen (H_2_) is considered to represent a clean, nontoxic, sustainable and high-density energy carrier^1^. However, the safe and economical storage, handling and transportation of H_2_ remains a challenge. FA (FA; HCOOH) is a promising H_2_ storage liquid, and has the advantages of low toxicity and a high hydrogen content (4.4 wt%), and so has received tremendous research interest^2,3^. H_2_ can be controllably released through the dehydrogenation of FA (Δ*G* = −32.8 kJ mol^−1^) at room temperature in the presence of a suitable catalyst^4,5^ and CO_2_-FA mediated H_2_ storage systems have been proposed^6,7^. The direct hydrogenation of CO_2_ to FA over a catalyst is considered to be an economical, environmentally friendly method of using FA to store H_2_, and benefits from the use of CO_2_ as a feedstock^8,9^. Therefore, there is currently a search for catalysts that promote both the hydrogenation of CO_2_ and the dehydrogenation of FA.

Recently, homogeneous metal complexes have proven to be efficient catalysts for both CO_2_ hydrogenation and FA dehydrogenation^7,10^. It has also been demonstrated that electron-rich metal centers can significantly enhance the catalytic activities of such materials^11,12^. Numerous studies have thus been dedicated to the fabrication of electron-rich active metal centers using electron-donating ligands, and high catalytic activities and selectivities have been reported^13^. Despite such achievements^14^, these catalysts remain difficult to recover and may exhibit poor stability, as well as the need for the concurrent use of organic solvents and harsh reaction conditions. Thus, it would be preferable to use heterogeneous catalysts for hydrogenation of CO_2_ to FA or for FA decomposition because these materials are easier to handle and to recover^15–17^. Recent studies have demonstrated that Pd nanoparticles represent one of the most active catalysts for CO_2_ hydrogenation to formate and for FA decomposition, affording considerable yields in the presence of additives^18,19^. The surface electron density of Pd nanoparticles has also been shown to significantly affect their catalytic activity^20^. Alloying Pd with other metals having different work functions so as to tailor the surface electron density of the Pd is emerging as a promising strategy for tuning the electron density of the Pd. PdAu^21^, PdAg^22,23^, PdCu^24^, PdAuCo^25^ and PdCuCr^26^ have all been synthesized and have shown enhanced catalytic activities as compared to monometallic Pd. In the present study, Ag was alloyed with Pd due to the low cost and high ductility of the former metal. Furthermore, considering that the electronegativities of Pd and Ag are 2.20 and 1.9, respectively, electron density should readily transfer from Ag atoms to Pd atoms, leading to the formation of electron-rich Pd, which plays an important role in CO_2_ hydrogenation as well as FA dehydrogenation^23,27^.

In general, metal nanoparticles can offer high surface-to-volume ratios and abundant active sites, as well as significantly enhanced activities as compared to larger particles^28^. However, these nanoparticles tend to have high surface energies and readily aggregate during catalytic reactions, especially upon heating, which significantly reduces their most helpful properties^29^. Consequently, considerable effort have been applied to the stabilization of metal nanoparticles. One promising strategy is to encapsulate metal nanoparticles inside metal organic frameworks (MOFs)^30^. MOFs, a class of porous coordination polymers, are built using metal ions as connecting centers and organic molecules as linkers, and have emerged as an attractive class of functional materials widely used in catalysis because of their high surface areas, increased porosities, permanent nanoscale cavities or open channels, and chemical diversity^31^. The use of MOFs as supports for metal nanoparticle catalysts should prevent agglomeration and detachment of the nanoparticles, thus preserving their intriguing properties in catalysis applications. There are two main approaches to incorporating metal nanoparticles in MOFs. The first, and most popular, method is the so-called “ship in a bottle” technique^32^, which involves the absorption of a metal precursor into previously formed porous materials followed by the reduction of the precursor to give metal nanoparticles. However, metal nanoparticles having a broad size range and unpredictable spatial distribution are inevitably formed on the external surfaces of the MOF. Another approach is termed “bottle around ship”^33,34^, and consists of the synthesis of individual surfactant-stabilized metal nanoparticles that are subsequently coated with the MOF. Although remarkable achievements have been reported in this field, a common and facile strategy to encapsulate metal nanoparticles in MOFs in conjunction with controllable spatial distributions and small particle sizes is still lacking.

**Figure 1:**
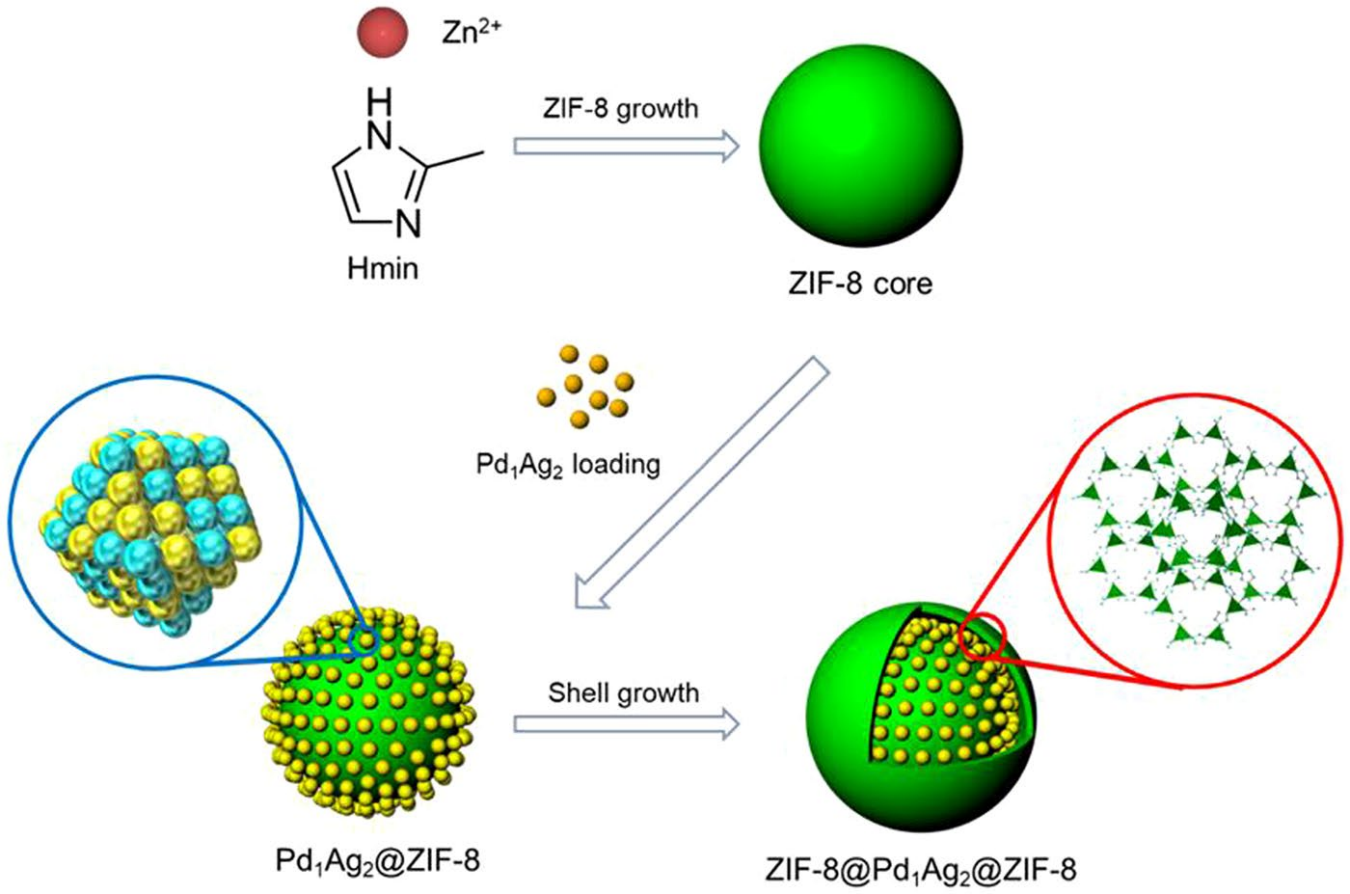
Schematic illustration of the ZIF-8@PdAg@ZIF-8 preparation process.

The zeolitic imidazolate framework (ZIF-8), having a zeolite-type structure with large cavities and small apertures, is well known for its chemical robustness and thermal stability, and can be synthesized at room temperature^35^. It has also been reported that metal nanoparticles supported on ZIF-8 show excellent catalytic activity and selectivity during FA dehydrogenation, possibly due to the presence of soft Lewis acid sites and functional groups capable of activating the FA^36^. To mitigate the aggregation of metal nanoparticles on the external surfaces of MOFs, as well as to prevent damage to MOFs during the post-reduction process, the present study developed a simple means of controllably encapsulating metal nanoparticles within ZIF-8. This “bottle around ship” approach involves the growth of a ZIF-8 core during an initial stage, using 2-methylimindazole (Hmin) moieties as organic linkers and Zn^2+^ ions as connecting centers, followed by the loading of PVP-stabilized PdAg nanoparticles onto the external surfaces of the ZIF-8 core, then coating of the nanoparticles with additional ZIF-8. Figure 1 illustrates the synthetic route for the controllable fabrication of the ZIF-8@PdAg@ZIF-8 catalyst. The composition and location of the incorporated PdAg alloy nanoparticles can be readily controlled via this process. The as-prepared ZIF-8@PdAg@ZIF-8 composite was examined as a catalyst for use in a hydrogen storage/production system based on FA. The PdAg alloy nanoparticles when confined by the crystallization process of the ZIF-8 afford a novel ZIF-8@PdAg@ZIF-8 catalyst that exhibits high activity and superior stability during the hydrogenation of CO_2_ to FA and the dehydrogenation of FA to CO_2_ and H_2_.

## Results and Discussion

**Figure 2:**
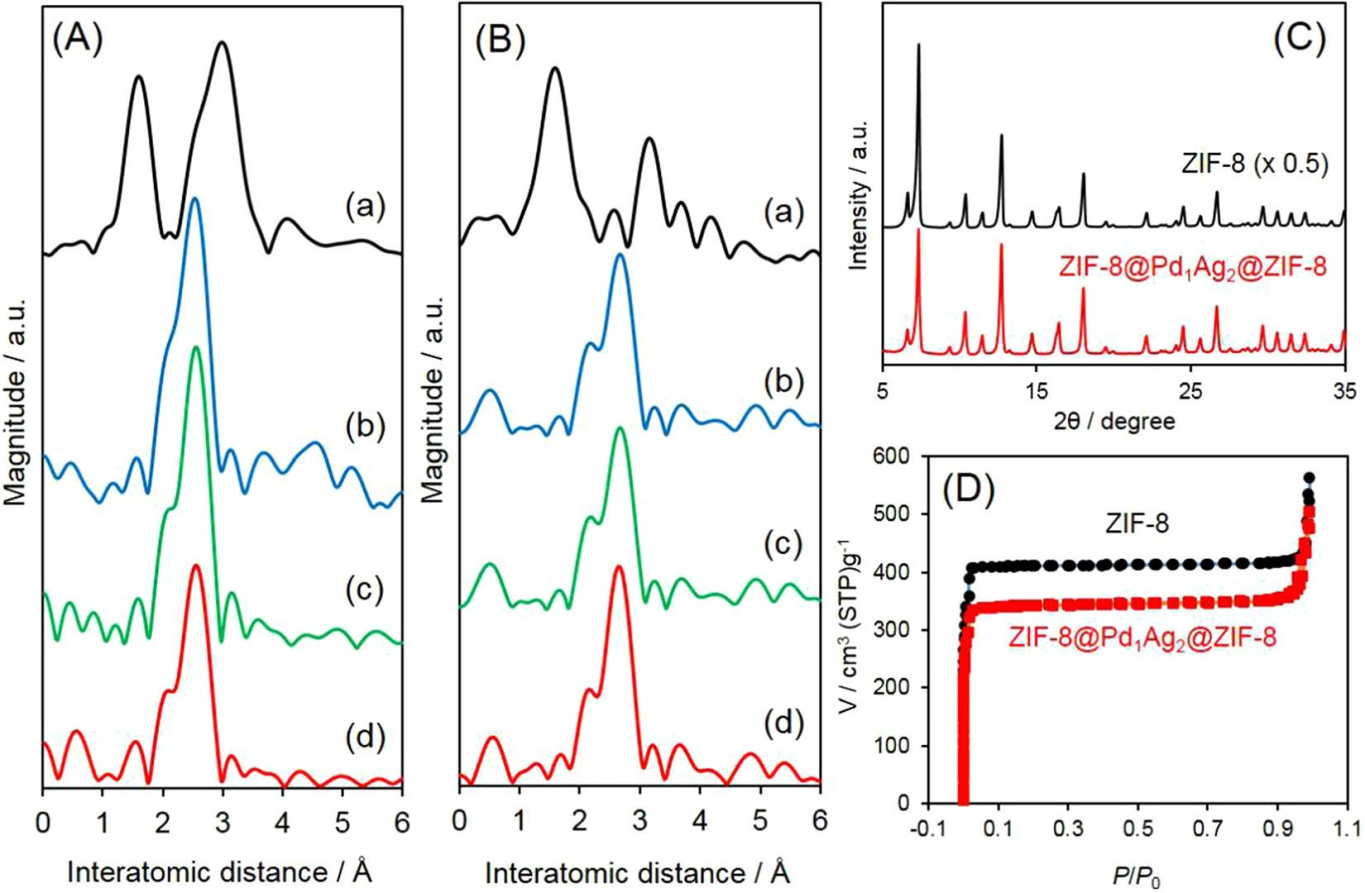
(**A**) Pd K-edge FT-EXAFS spectra of ((a) PdO, (b) Pd foil, (c) Pd_1_Ag_2_, (d) ZIF-8@Pd_1_Ag_2_@ZIF-8), (**B**) Ag K-edge FT-EXAFS spectra of ((a) AgO, (b) Ag foil, (c) Pd_1_Ag_2_, (d) ZIF-8@Pd_1_Ag_2_@ZIF-8), (**C**) XRD patterns, and (**D**) N_2_-adsorption/desorption isotherms.

Bimetallic Pd_1_Ag_2_ nanoparticles were successfully synthesized using a non-aqueous method. As shown in Fig. S1, well-dispersed Pd_1_Ag_2_ nanoparticles were obtained. These nanoparticles displayed a very narrow size distribution, as demonstrated by Fig. S1b, and no agglomeration was observed. The average diameter was determined to be 2.2 nm. The alloy structure was assessed by examining the chemical environments of the Pd and Ag species by spectroscopic methods. Figure 2A displays the Fourier transform (FT) of *k*^3^-weighted extended X-ray absorption fine structure (EXAFS) data at the Pd K-edge of the Pd_1_Ag_2_ solution, as well as results for reference samples. In the case of Pd foil, the adjacent Pd–Pd bonds in the metallic form generated a single peak at approximately 2.5 Å, while two peaks were produced by the PdO, ascribed to the Pd–O shell and the Pd–O–Pd shell^37^. Similar to the Pd foil, the Pd_1_Ag_2_ solution and the ZIF-8@Pd_1_Ag_2_@ZIF-8 generated a main peak corresponded to metallic Pd–Pd bonding. However, the Pd–Pd distance was slightly longer as compared to the foil, demonstrating the formation of heteroatomic bonding in the Pd_1_Ag_2_ nanoparticles^20^. In the case of the Ag K-edge FT-EXAFS spectra, the Pd_1_Ag_2_ nanoparticles, ZIF-8@Pd_1_Ag_2_@ZIF-8 and Ag foil also showed a single peak at approximately 2.67 Å, and the interatomic distance in the Pd_1_Ag_2_ nanoparticles encapsulated in the ZIF-8 was shorter than that in the Ag foil. Thus, the formation of heteroatomic Pd–Ag bonding was confirmed (Fig. 2B)^20^. In addition, a peak corresponding to the Ag–O bond (such as that generated by AgO at approximately 1.7 Å) is absent. On the basis of the above results, it is reasonable to assume that the Pd_1_Ag_2_ nanoparticles had an alloy structure.

The coordination numbers (CNs) and bond lengths (*R*) of the ZIF-8@Pd_1_Ag_2_@ZIF-8 sample as determined from EXAFS curve fitting are presented in Table S1. The data were well fitted using not only Pd−Pd and Ag−Ag bonds but also heteroatomic Pd−Ag bonds. The Pd−Pd bond distances were evidently shorter than those of Pd−Ag bonds, which in turn were shorter than Ag−Ag bonds. These results are consistent with the shifts observed in the main peaks of the FT-EXAFS spectra due to metallic bonding. It is widely accepted that the atoms inside an fcc lattice have a CN of 12. The average diameter of the encapsulated PdAg was 2.8 nm whose total CN is less than 12. Because the proportion of Pd in the nanoparticles was less than that of Ag (Pd:Ag = 1:2), the CN_total_ (CN_Pd−Pd_ + CN_Pd−Ag_) at the Pd K-edge was lower than that (CN_Ag−Ag_ + CN_Ag−Pd_) at the Ag K-edge.

Crystallographic information regarding the samples was obtained by X-ray diffraction (XRD) analysis, with the results shown in Fig. 2C. The diffraction pattern of the pure ZIF-8 is in good agreement with a reported simulated pattern, demonstrating the successful fabrication of this material^38^. The ZIF-8@Pd_1_Ag_2_@ZIF-8 sample also generated a diffraction pattern similar to that of the pure ZIF-8, suggesting that the encapsulation of Pd_1_Ag_2_ nanoparticles within the ZIF-8 did not change the framework structure. However, the intensities of the diffraction peaks were weaker than those of the pure ZIF-8, presumably because the encapsulated Pd_1_Ag_2_ nanoparticles introduced some disorder into the MOF crystal. In addition, diffraction peaks assignable to Pd and Ag do not appear in the catalyst pattern, possibly because of the low concentrations of these elements and the small particle size. The pore structures of the samples were characterized using the N_2_ sorption technique. As shown in Fig. 2D, the pure ZIF-8 produced a type-I isotherm that was completely reversible, which is typical of microporous materials. The Brunauer-Emmett-Teller (BET) surface area determined by N_2_ adsorption-desorption for this material was 1110 m^2^ g^−1^. The Pd_1_Ag_2_ nanoparticles encapsulated in ZIF-8 produced a similar isotherm to that of pure ZIF-8 except for a slight decrease in the N_2_ uptake, suggesting a decrease in the number of micropores after the encapsulation of the Pd_1_Ag_2_ nanoparticles. This resulted in a slight decrease in the surface area to 926.3 m^2^ g^−1^. Based on the XRD and BET analyses, it appears that the crystallinity and porosity of the ZIF-8 are well preserved after Pd_1_Ag_2_ encapsulation.

**Figure 3:**
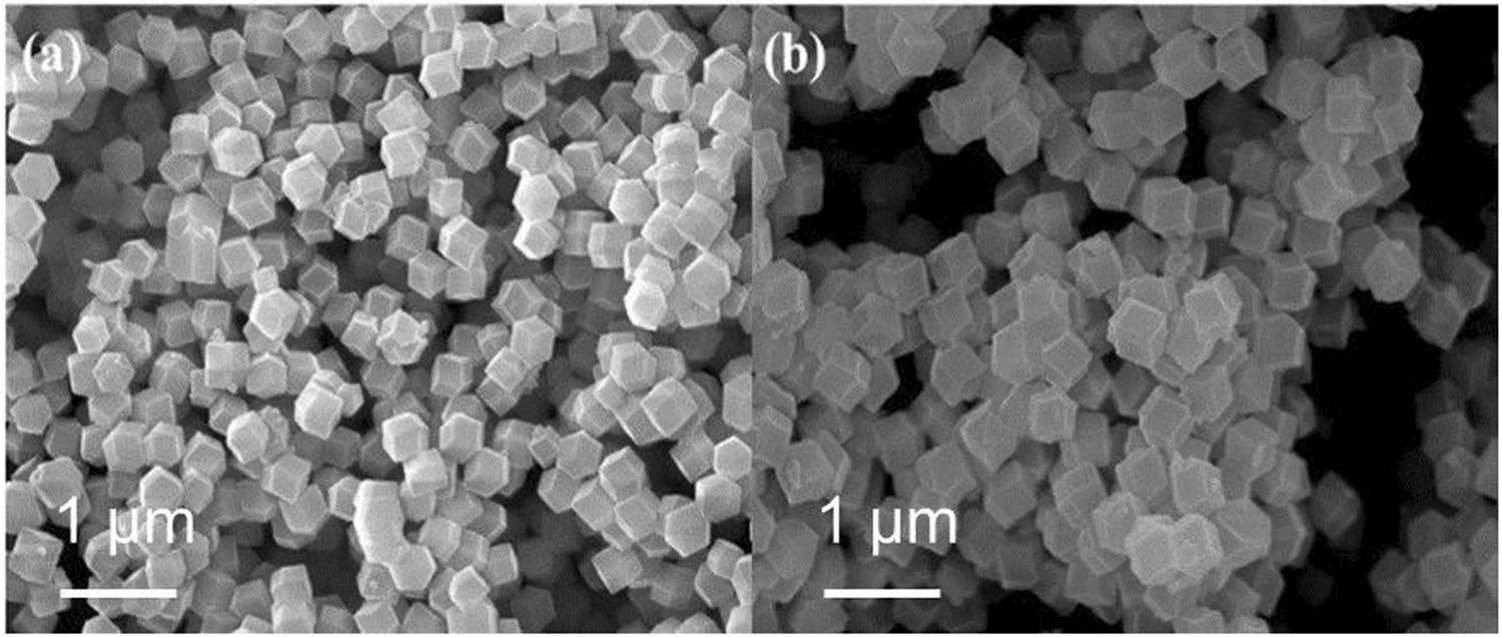
SEM images of (**a**) pure ZIF-8 and (**b**) ZIF-8@Pd_1_Ag_2_@ZIF-8.

**Figure 4:**
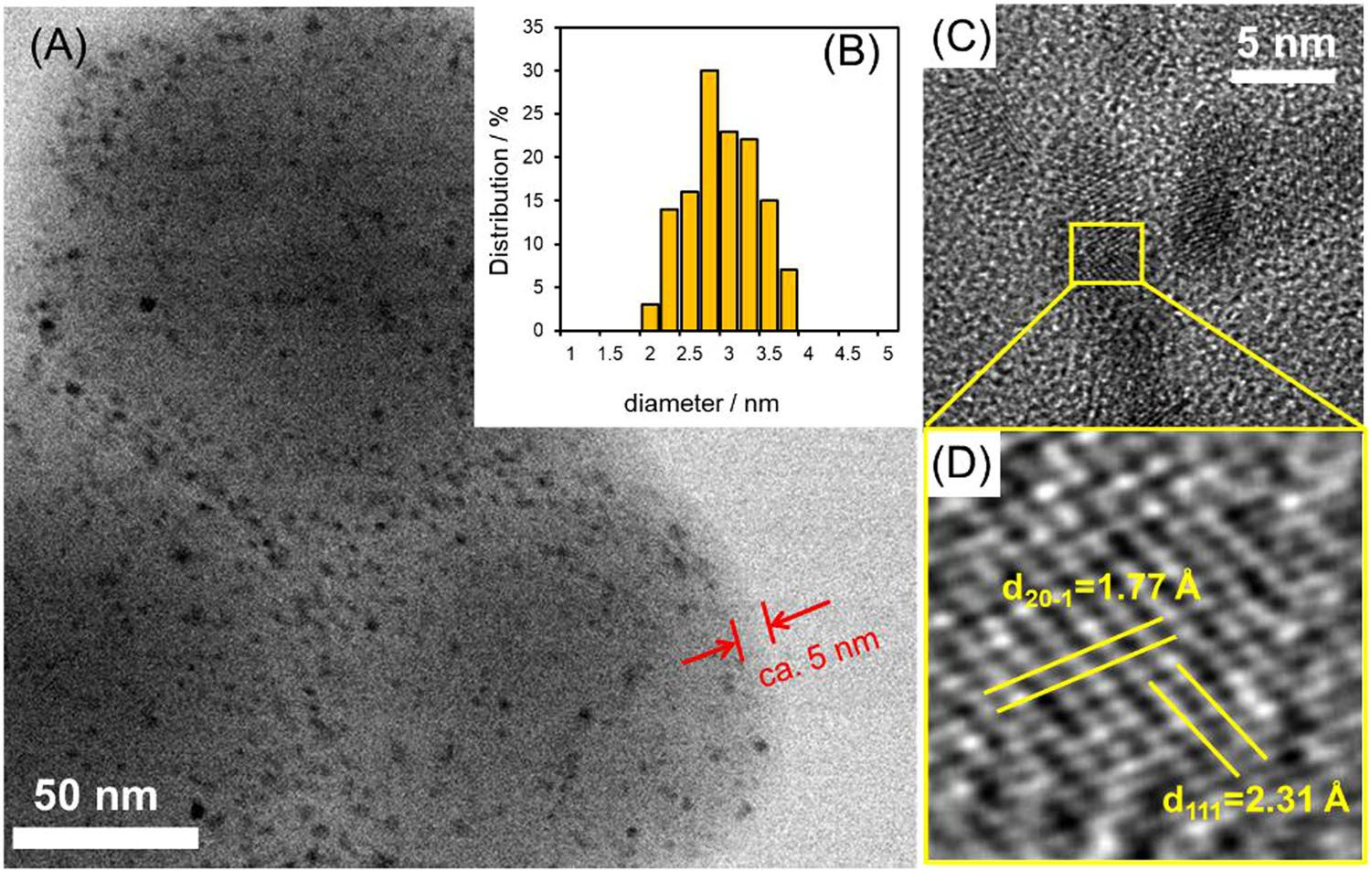
(**A**) TEM image, (**B**) size distribution diagram, and (**C**,**D**) HR-TEM images of ZIF-8@Pd_1_Ag_2_@ZIF-8.

The morphologies of the pure ZIF-8 and the ZIF-8@Pd_1_Ag_2_@ZIF-8 are shown in Fig. 3. The pure ZIF-8 had a rhombic dodecahedral morphology in conjunction with a particle size of approximately 350 nm. The rhombic dodecahedra were also uniformly dispersed without any significant aggregation. The morphology of the ZIF-8@Pd_1_Ag_2_@ZIF-8 did not undergo any obvious changes from that of the pure ZIF-8. The size and spatial distribution of the Pd_1_Ag_2_ nanoparticles were assessed by transmission electron microscopy (TEM), and the results are summarized in Fig. 4. Pd_1_Ag_2_ nanoparticles covered by a thin shell of ZIF-8 can be clearly observed, with the shell having a thickness of approximately 5 nm, as shown in Figs 4A and S2. The average size of the encapsulated Pd_1_Ag_2_ nanoparticles was determined to be 2.8 nm and these nanoparticles had a very narrow size distribution (Fig. 4B). In addition, no Pd_1_Ag_2_ nanoparticles were deposited on the external surface of the ZIF-8@Pd_1_Ag_2_@ZIF-8. The high-resolution TEM (HRTEM) image clearly shows the (111) and (20-1) planes, with lattice spacings of 2.31 and 1.77 Å (Fig. 4C,D), respectively. It should be noted that the lattice spacing of the (111) plane of Pd_1_Ag_2_ is smaller than that of the (111) plane of Ag, but larger than that of the (111) plane of Pd, while the lattice spacing of the (20-1) plane of Pd_1_Ag_2_ is between those of the (20-1) planes of Pd and Ag (see Table S2 for details). These results provide further evidence that the Pd_1_Ag_2_ nanoparticles had a true alloy structure^36^.

**Figure 5:**
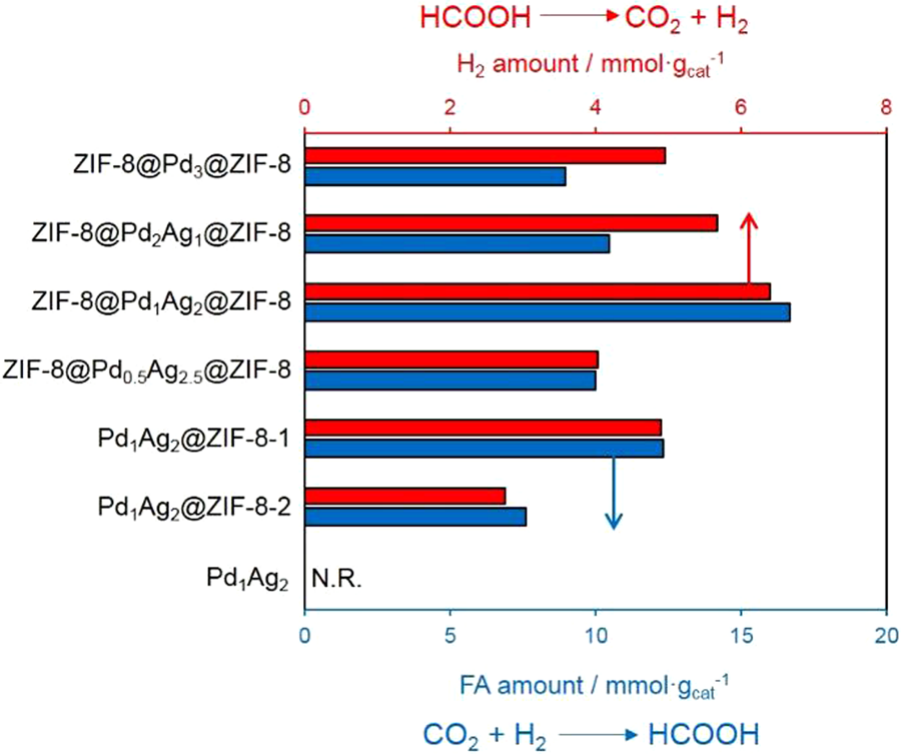
Comparison of catalytic activities during the hydrogenation of CO_2_ to produce FA (blue bars) and dehydrogenation of FA (red bars) over various catalysts.

The ZIF-8@PdAg@ZIF-8 catalyst was subsequently applied to the hydrogenation of CO_2_ to produce FA. The reaction was carried out in a stainless steel reactor containing a 1 M aqueous NaHCO_3_ solution (10 mL) at 100 °C for 24 h under a total pressure of 2.0 MPa (H_2_:CO_2_ = 1:1). Various catalysts were prepared by varying the molar ratio of Ag to Pd from 0:3 to 2.5:0.5 to determine the optimal composition, and Fig. 5 summarizes the catalytic activities over the different catalysts. No reaction occurred on the unsupported Pd_1_Ag_2_ nanoparticles when using the same amount of Pd_1_Ag_2_ as was encapsulated within the ZIF-8. This negative result occurred because the unsupported Pd_1_Ag_2_ nanoparticles aggregated into larger particles under the catalytic reaction conditions, as evidenced by the TEM image after the reaction (Fig. S3); the surface energy increases as the particle size decreases, which frequently leads to serious aggregation of the ultra-fine particles in order to minimize the total surface energy^39^. This phenomenon demonstrates the importance of ZIF-8 in preventing the aggregation of Pd_1_Ag_2_ nanoparticles during the catalytic reaction. On the other hand, we can still observe the small amount of colloidal Pd_1_Ag_2_ nanoparticles with a mean diameter of ca. 2 nm. This results suggest that stabilization of PdAg nanoparticles with strongly binding PVP ligands was inactive because of the prevention of the interaction with reactants^40^. It is interesting to observe that, in each case, Pd_1_Ag_2_ nanoparticles associated with ZIF-8 exhibited higher catalytic activity than that of ZIF-8@Pd_3_@ZIF-8, demonstrating that the PdAg alloy nanoparticles have a positive effect on the catalytic reaction. The catalytic activities of the various ZIF-8@PdAg@ZIF-8 samples were also highly dependent on the composition of the PdAg nanoparticles, and the optimal Pd:Ag ratio was found to be 1:2. The ZIF-8@Pd_1_Ag_2_@ZIF-8 thus exhibited the highest catalytic activity among all samples, with a value of 16.68 mmol g_(catal.)_^−1^ after 24 h. This level of activity was almost twice that obtained over ZIF-8@Pd_3_@ZIF-8 under the identical reaction conditions. Thus, decreasing the Pd:Ag ratio from 3:0 to 1:2 increased the catalytic activity. It has been widely reported that electron-rich metal centers by alloying with Ag can significantly enhance catalytic activity during FA dehydrogenation^20,41^. However, further decreases in the Pd:Ag ratio lowered the catalytic activity due to a decrease in the number of active Pd sites.

**Figure 6:**
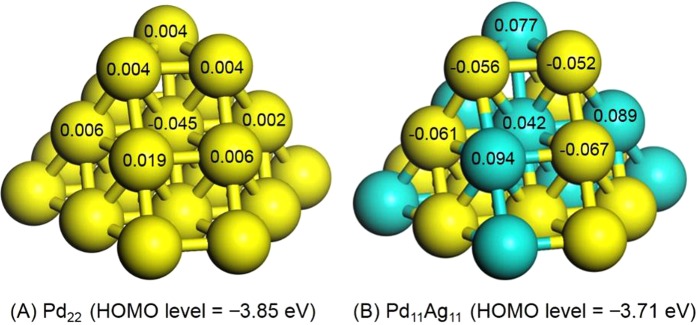
Representative Mulliken atomic charges and HOMO levels determined by DFT calculations for (**A**) Pd_22_ and (**B**) Pd_11_Ag_11_ clusters.

As noted, charge was transferred from the Ag atoms to the Pd atoms because the electronegativities of Pd and Ag are 2.20 and 1.9, respectively. Density functional theory (DFT) calculations employing Pd_11_ and Pd_11_Ag_11_ clusters as models of monometallic and alloy nanoparticles confirmed that the Pd atoms in the Pd_11_Ag_11_ clusters were indeed negatively charged in comparison with those in a Pd_11_ cluster, while the Ag atoms were positively charged as a result of charge transfer (Fig. 6). Additionally, the DFT results demonstrated that the highest occupied molecular orbital (HOMO) of the Pd_11_Ag_11_ cluster had a greater energy level than that of the monometallic Pd_22_ cluster, with values of −3.85 and −3.71 eV, respectively. This elevated HOMO level would be expected to increase the electron-richness of the active Pd atoms.

**Figure 7:**
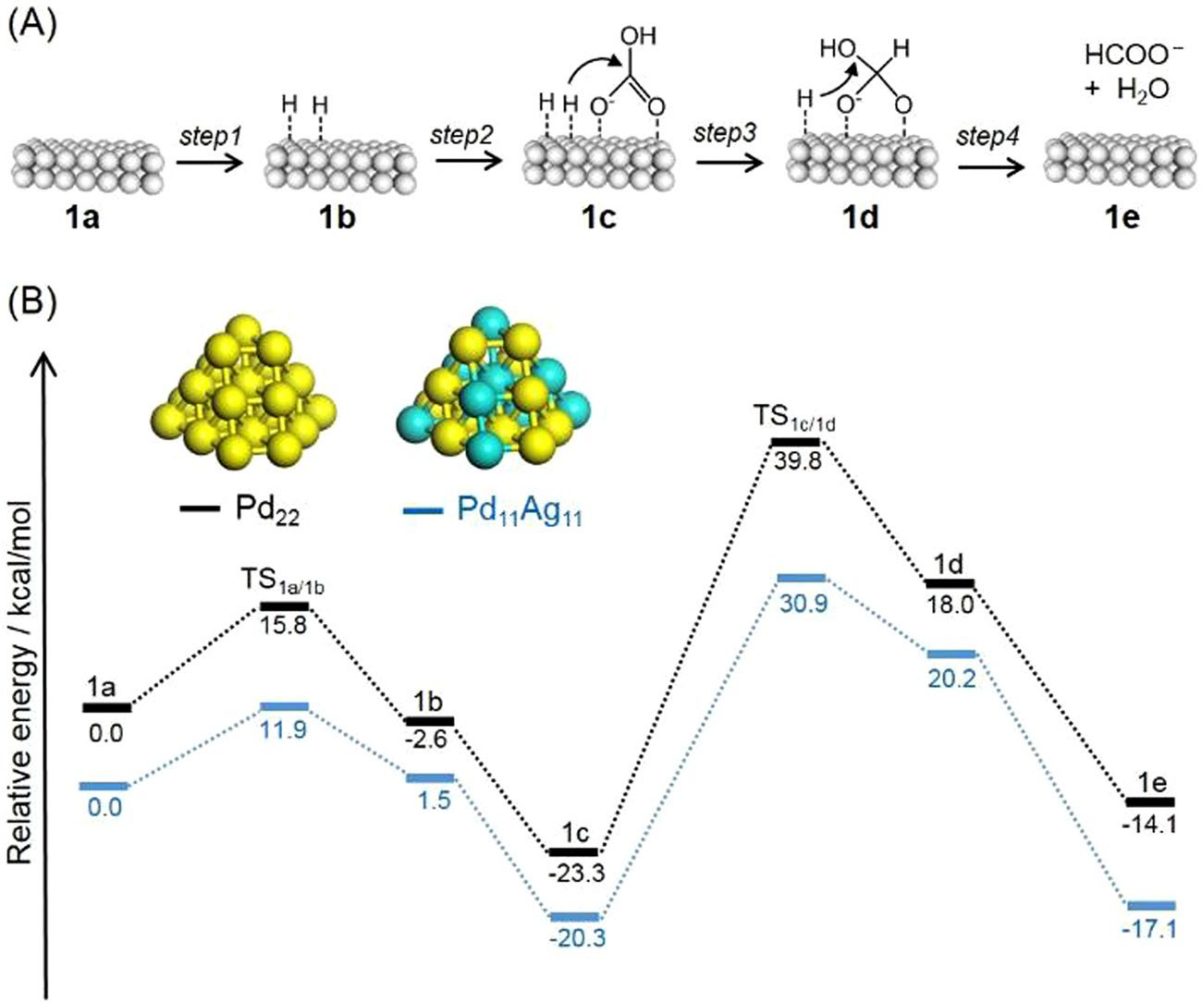
(**A**) A possible reaction pathway and (**B**) potential energy profiles for CO_2_ hydrogenation to FA over Pd_22_ and Pd_11_Ag_11_ cluster models.

Figure 7A displays a proposed mechanism for CO_2_ hydrogenation, initiated by the dissociation of H_2_ at a Pd atom to afford Pd-hydride (**1b**) (step 1). This is followed by the adsorption of bicarbonate (HCO_3_^−^) generated by the dissolution of CO_2_ in water (step 2). The resulting reaction intermediate (**1c**) undergoes hydrogenation by a neighboring hydride species to give another intermediate **1d** (step 3). Finally, the production of formate together with H_2_O (step 4) completes the catalytic cycle^22,23,42^. In an effort to elucidate the cause of the positive effect of alloying with Ag, potential energy profiles were produced using DFT calculations, employing Pd_22_ and Pd_11_Ag_11_ model clusters (Fig. 7B). In the case of Pd_22_, the H_2_ dissociation energy via transition state TS_1a/1b_ was calculated to be 15.8 kcal/mol. After the adsorption of HCO_3_^−^, hydrogenation by the neighboring hydride species occurs via TS_1c/1d_ with an energy barrier of 63.1 kcal/mol. The energy barrier in step 4, in which formate is spontaneously produced along with H_2_O when the OH of the HCO_3_^−^ is attacked by another Pd-hydride species, is quite low. These results indicate that step 3 is the rate-limiting stage in the present catalytic cycle. In the case of the Pd_11_Ag_11_ cluster model, the activation energy for the dissociation of H_2_ was determined to be 11.9 kcal/mol, while the reduction of HCO_3_^−^ was found to be occur with a barrier of 51.2 kcal/mol. These results indicate that alloying with Ag plays an important role in promoting the rate-limiting step 3 rather than the H_2_ dissociation step 1.

The catalytic activities of Pd_1_Ag_2_-loaded ZIF-8 (Pd_1_Ag_2_@ZIF-8) samples without a core-shell structure and prepared by different deposition methods were also investigated. As shown in Fig. 5, poor catalytic performance was obtained from the Pd_1_Ag_2_@ZIF-8-1 and Pd_1_Ag_2_@ZIF-8-2, due to the relatively large Pd_1_Ag_2_ nanoparticles size, which had mean sizes of 10.5 and 7.6 nm, respectively (Fig. S4). In comparison, the ZIF-8@Pd_1_Ag_2_@ZIF-8 exhibited improved catalytic activity, which can be ascribed to the high degree of dispersion of the Pd_1_Ag_2_ nanoparticles within the ZIF-8 as well as the positive effect of the thin shell protecting the nanoparticles during the reaction process. A TEM image of the ZIF-8@Pd_1_Ag_2_@ZIF-8 after the reaction is shown in Fig. S5. The Pd_1_Ag_2_ nanoparticles evidently remained well dispersed within the ZIF-8, with no significant aggregation, confirming the remarkable stability of this catalyst. To further assess the stability of the ZIF-8@Pd_1_Ag_2_@ZIF-8, the catalyst was recovered from the reaction solution using centrifugation and washed with water. The recycled ZIF-8@Pd_1_Ag_2_@ZIF-8 could be re-used at least three times without a significant loss of activity, as demonstrated in Fig. S6. Based on the above results, it is clear that the present synthetic approach avoids the typical issue of the aggregation of metal nanoparticles on the external surfaces of MOFs as well as prevents damage to the MOF during the post-reduction process. Consequently, the catalytic activity during CO_2_ hydrogenation to produce FA is enhanced.

Figure 5 also displays the catalytic activity of the ZIF-8@Pd_1_Ag_2_@ZIF-8, as well as those of unsupported Pd_1_Ag_2_ nanoparticles and Pd_1_Ag_2_-loaded ZIF-8 samples (Pd_1_Ag_2_/ZIF-8-1 and Pd_1_Ag_2_/ZIF-8-1) prepared by different methods during the dehydrogenation of FA to produce H_2_. These results are well correlated with the extent of hydrogenation of CO_2_ to FA over these same materials. No reaction occurred using the unsupported Pd_1_Ag_2_ nanoparticles due to the significant aggregation of these nanoparticles in the presence of the FA, as shown in Fig. S7, indicating the importance of the support material to prevent the agglomerations. Again, we can still observe the small amount of colloidal Pd_1_Ag_2_ nanoparticle while keeping its original particles size, suggesting that strongly binding PVP ligands prevents the reaction of FA decomposition. Considerable activity was exhibited by the Pd_1_Ag_2_@ZIF-8-1, and this activity was enhanced in the case of the ZIF-8@Pd_1_Ag_2_@ZIF-8 due to the high dispersion of the Pd_1_Ag_2_ nanoparticles within the ZIF-8 and protection of the nanoparticles from agglomeration by the shell.

**Figure 8:**
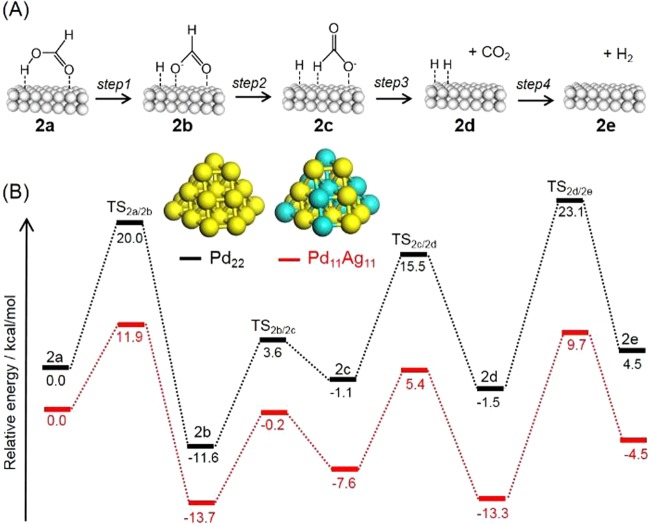
(**A**) A possible reaction pathway and (**B**) potential energy profiles for dehydrogenation to FA over Pd_22_ and Pd_11_Ag_11_ cluster models.

To verify the positive effect of the alloy in terms of promoting the FA dehydrogenation, potential energy profiles were calculated based on DFT, and are summarized in Fig. 8. The lowest energy FA adsorption structure was the *trans*-M(O)–M(H-O)-bridged configuration (**2a**, M = Pd or Ag). The HCOOH adsorbed on the Pd_11_ cluster model evidently undergoes O–H bond cleavage to produce the formate intermediate **2b** via TS_2a/2b_ with an energy barrier of 20.0 kcal/mol (step 1). Subsequently, this species is isomerized to afford a *trans*-M(H)–Pd(O)-bridged HCOOH configuration structure (**2c**) via TS_2b/2c_, with a barrier of 15.2 kcal/mol (step 2). The reaction intermediate **2c** then undergoes C–H bond cleavage to form CO_2_ and a Pd–H species (**2d**) via TS_2c/2d_ with a barrier of 16.6 kcal/mol (step 3). Following this, the catalytic cycle is completed by H_2_ release via TS_2d/2e_ with a barrier of 24.6 kcal/mol (step 4). The activation energies for each elementary steps over the Pd_11_Ag_11_ cluster model were determined to be 11.9, 13.5, 13.0 and 23.0 kcal/mol for steps 1–4, respectively. Alloying Pd with Ag changes the electron density at the active Pd sites due to charge transfer from Ag to Pd resulting from the different work functions of the two elements. This boosts the intrinsic catalytic performance of Pd for FA dehydrogenation^12,20,22,23^.

The kinetic isotopic effect was assessed using HCOOH and DCOOH, and the *k*_H_/*k*_D_ value obtained with ZIF-8@Pd_1_Ag_2_@ZIF-8 (*k*_H_/*k*_D_ = 2.7) was found to be smaller than that associated with the ZIF-8@Pd_3_@ZIF-8 (*k*_H_/*k*_D_ = 3.4), suggesting that C-H dissociation is facilitated by the presence of electron-rich Pd arising from the charge transfer from Ag to Pd (Table S3). This role of electron-rich Pd atoms in achieving high catalytic activity has also been reported previously^20,43^. Interestingly, the *k*_H_/*k*_D_ values were found to be 1.3 and 1.25 for the ZIF-8@Pd_3_@ZIF-8 and ZIF-8@Pd_1_Ag_2_@ZIF-8, respectively. These small values can be ascribed to the positive effect of N atoms within the ZIF-8 framework, which serve as proton scavengers to facilitate the dissociation of the O-H bond in FA to produce a formate intermediate^20,41,44,45^.

## Conclusions

Bimetallic PdAg nanoparticles were successfully encapsulated in ZIF-8 using a “bottle around ship” approach with the aim to prevent the aggregation of the PdAg nanoparticles during the catalytic reaction. For the first time, ZIF-8@PdAg@ZIF-8 was applied to the hydrogenation of CO_2_ to FA and the dehydrogenation of FA to CO_2_ and H_2_. Among investigated, ZIF-8@Pd_1_Ag_2_@ZIF-8 exhibited the highest catalytic performance for both reactions. Higher or lower Pd:Ag ratios decreased the catalytic activity, demonstrating the optimum relative proportion of both elements in the nanoparticles. The enhanced catalytic performance of this material can be explained by the synergistic effect of combining the PdAg alloy and ZIF-8 support. The electron-rich Pd sites caused by charge transfer from Ag to Pd as well as the basic N groups within the nanopores of the ZIF-8 play important roles in promoting the formation of FA and H_2_. Poor catalytic performance was obtained by the Pd_1_Ag_2_@ZIF-8 due to the formation of relatively large nanoparticles, which confirms the importance of the core-shell structure. Moreover, the catalyst was stable and reusable because the outer shell made of composite prevented the aggregation of the PdAg nanoparticles during the catalytic reaction, such that there was no significant loss of catalytic activity after several recycling trials. The present study may pave the way to the development of practical and environmentally benign reversible hydrogen storage systems based on FA.

## Experimental Section

### Synthesis of Pd_*n*_Ag_*m*_ nanoparticles

Pd_*n*_Ag_*m*_ nanoparticles (*n* and *m* represents the theoretical mole ratio between Pd and Ag) were synthesized by using a reported method with a small modification^46^. In a typical experiment, two solutions were prepared. Taking the Pd_1_Ag_2_ as an example, solution 1 (containing 0.416 g of polyvinylpyrrolidone (PVP, 30 K) as stabilizing agent, 0.0424 g of AgNO_3_ as Ag precursor and 15 mL ethylene glycol as solvent) was stirred at 80 °C for 2 h. Solution 2 (containing 0.02805 g of Palladium (II) acetate as Pd precursor and 6.25 mL of 1,4-Dioxane as solvent) was stirred at room temperature. Solution 1 was cooled to 0 °C and then 0.7 mL of 1 M NaOH solution was added to adjust the pH of the resulting mixture under stirring. Then, solution 2 was poured into solution 1 under vigorous stirring and the final mixture was heated up to 100 °C. for 2 h. After the metallic colloid preparation, PdAg nanoparticles were purified by adding an excess of acetone and shaking the solution, which caused the extraction of PVP to the acetone phase and flocculation of the PdAg nanoparticles. The supernatant organic phase was removed and the purified nanoparticles were redispersed in 40 mL of methanol (Pd_1_Ag_2_: 0.375 mmol). Meanwhile, 0.375 mmol of Pd_3_, Pd_2_Ag_1_ and Pd_0.5_Ag_2.5_ nanoparticles dissolved in 40 mL were synthesized with same procedure.

### Synthesis of ZIF-8@PdAg@ZIF-8

In a typical experimental, 0.037 g of Zn(NO_3_)_2_ and 0.0103 g of Hmin were respectively dissolved in 10 mL of methanol, the mixture was allowed to react at room temperature for 30 min without stirring, then 0.5 mL (Pd_1_Ag_2_: 9.4 μmol) of pre-synthesized PdAg nanoparticles solution was added into above mixture, further keep at room temperature without stirring. After 24 h, the product was collected by centrifugation, washed several times with methanol. Finally, the products were dried under vacuum overnight. The obtained sample is noted as ZIF-8@Pd_1_Ag_2_@ZIF-8. The loaded Pd amount was determined to be 2.98 wt.% by ICP analysis. Meanwhile, ZIF-8@Pd_3_@ZIF-8, ZIF-8@Pd_2_Ag_1_@ZIF-8 and ZIF-8@Pd_0.5_Ag_2.5_@ZIF-8 were synthesized with same synthetic route. For comparison, the added metal nanoparticles amount was same as ZIF-8@Pd_1_Ag_2_@ZIF-8.

### Synthesis of Pd_1_Ag_2_@ZIF-8-1

Pd_1_Ag_2_ loaded on the external surface of ZIF-8 (noted as Pd_1_Ag_2_@ZIF-8-1) were synthesized by introducing the Pd_1_Ag_2_ with the intention of 2.98 wt.% of Pd in the methanol and ZIF-8 suspension, followed by stirring at room temperature for 6 hours, then the product was collected under vacuum condition at 60 °C.

### Synthesis of Pd_1_Ag_2_@ZIF-8-2

Pd_1_Ag_2_@ZIF-8-2 was synthesized by impregnation method. Typically, 0.2 g of ZIF-8 was dispersed in 20 mL of methanol and sonicated for 15 min. then, a given amount of PdCl_2_ (with the intention of 2.98 wt.% of Pd) and AgNO_3_ aqueous solution was added and stirred for 6 hours at ambient temperature. After stirring, NaBH_4_ (ten times molar ratio of loading metal) was added into above solution and further stirred for 15 min. Finally, the product was collected by under vacuum condition at 60 °C.

## Characterization

Nitrogen adsorption studies were performed by using BEL-SORP max system (BEL Japan, Inc.) at 77 K. In order to remove the adsorbed impurities, the samples were degassed in vacuum at 473 K for 24 h prior to analysis. Powder X-ray diffraction (XRD) measurements were conducted by using a RigakuRINT2500 Ultima IV X-ray diffractometer with Cu Kα radiation (λ = 1.5406 Å). TEM micrographs were obtained by using Hitachi Hf-2000 field emission-transmission electron microscope (FE-TEM) equipped with Kevex energy-dispersive X-ray detector operated at 200 kV. Pd and Ag K-edge X-ray Absorption Fine Structure (XAFS) were performed using a fluorescence-yield collection technique at the BL01B1 station with an attached Si (111) monochromator at SPring-8, JASRI, Harima, Japan (Prop. No. 2017A1063, 2017A1057). The EXAFS data were normalized by fitting the background absorption coefficient, around the energy region higher than that of the edge of about 35–50 eV, with smooth absorption of an isolated atom. Fourier transformation (FT) of *k*^3^-weighted normalized EXAFS data was performed over the range of 3.0 Å < *k*/Å^−1^ < 12 Å to obtain the radial structure function. Backscattering amplitude and phase shift parameters for a curve-fitting analysis were theoretically calculated with FEFF8.40 code. DFT calculations were performed with the DMol^3^ program in Materials Studio 17.2^47,48^. The generalized gradient approximation (GGA) exchange-correlation functional proposed by Perdew, Burke, and Ernzerhof (PBE) was combined with the double-numerical basis set plus polarization functions (DNP). The top layers of Pd_11_ and Pd_11_Ag_11_ cluster models were allowed to relax during geometry optimizations, while the bottom two layers of were fixed at the corresponding bulk position.

### Hydrogenation of CO_2_

The catalytic activity test for hydrogenation of CO_2_ to FA was performed in an aqueous solution. Briefly, a sample (50 mg) was suspended in NaHCO_3_ aqueous solution (10 mL, 1 M) in an autoclave, and the pressure was increased to 1.0 MPa of CO_2_ and then increased to 2.0 MPa with H_2_. The reaction system was heated to 373 K and stirred for 24 h. The FA was analysed by HPLC with a Shimazu HPLC instrument equipped with a Bio-radAminerganic Analysis Column and an Aminex HPX-87H Ion Exclusion Column. The mobile phase is H_2_SO_4_ (5 mM, 0.500 mL/min). The catalytic performance, defined as the mmol of formic acid produced per gram of whole catalyst, was calculated on the basis of the whole catalyst amount and by using the amount of formic acid generated after 24 h.

### Dehydrogenation of FA

The catalytic behavior of the ZIF-8@Pd_1_Ag_2_@ZIF-8 for dehydrogenation of FA was evaluated by using a closed liquid-phase system. 20 mg of each powder samples and 4.8 mL of methanol were mixed into a Pyrex reaction vessel (30 mL) which was sealed with a rubber septum. Afterward, samples were treated in an ultrasound bath for 15 min to disperse the catalysts. Then, the mixture was bubbled with argon gas for 30 min to ensure inert atmosphere. Subsequently, 0.2 mL of FA was added into the vessel with magnetic stirring at oil bath (30 °C). The H_2_ evolution was monitored by using a Shimadzu GC14B equipped with MS5A column after fixed reaction times. The catalytic performance, defined as the mmol of H_2_ produced per gram of whole catalyst, was calculated on the basis of the whole catalyst amount and by using the amount of H_2_ generated after 2 h.

## Supplementary information

Supporing Inforamtion

## References

[1] Yang J, Sudik A, Wolverton C, Siegel DJ (2010). High Capacity Hydrogen Storage Materials: Attributes For Automotive Applications And Techniques For Materials Discovery. Chem. Soc. Rev..

[2] Grasemann M, Laurenczy G (2012). Formic Acid As A Hydrogen Source - Recent Developments And Future Trends. Energy Environ. Sci..

[3] Singh AK, Singh S, Kumar A (2016). Hydrogen Energy Future With Formic Acid: A Renewable Chemical Hydrogen Storage System. Catal. Sci. Tech..

[4] Boddien A (2010). Iron-Catalyzed Hydrogen Production from Formic Acid. J. Am. Chem. Soc..

[5] Mellmann D, Sponholz P, Junge H, Beller M (2016). Formic Acid As A Hydrogen Storage Material - Development Of Homogeneous Catalysts For Selective Hydrogen Release. Chem. Soc. Rev..

[6] Hull JF (2012). Reversible Hydrogen Storage Using Co2 And A Proton-Switchable Iridium Catalyst In Aqueous Media Under Mild Temperatures And Pressures. Nature Chem..

[7] Enthaler S, von Langermann J, Schmidt T (2010). Carbon Dioxide And Formic Acid-The Couple For Environmental-Friendly Hydrogen Storage?. Energy Environ. Sci..

[8] Bi QY (2014). An Aqueous Rechargeable Formate-Based Hydrogen Battery Driven by Heterogeneous Pd Catalysis. Angew. Chem. Int. Ed..

[9] Mori K, Taga T, Yamashita H (2017). Isolated Single-Atomic Ru Catalyst Bound on a Layered Double Hydroxide for Hydrogenation of CO_2_ to Formic Acid. ACS Catal..

[10] Filonenko GA, van Putten R, Schulpen EN, Hensen EJM, Pidko EA (2014). Highly Efficient Reversible Hydrogenation of Carbon Dioxide to Formates Using a Ruthenium PNP-Pincer Catalyst. ChemCatChem.

[11] Wang WH (2014). Formic Acid Dehydrogenation with Bioinspired Iridium Complexes: A Kinetic Isotope Effect Study and Mechanistic Insight. ChemSusChem.

[12] Tedsree K (2011). Hydrogen Production From Formic Acid Decomposition At Room Temperature Using a Ag-Pd Core-Shell Nanocatalyst. Nature Nanotech..

[13] Wang WH, Hull JF, Muckerman JT, Fujita E, Himeda Y (2012). Second-Coordination-Sphere And Electronic Effects Enhance Iridium(Iii)-Catalyzed Homogeneous Hydrogenation Of Carbon Dioxide In Water Near Ambient Temperature And Pressure. Energy Environ. Sci..

[14] Akbayrak S, Tonbul Y, Ozkar S (2017). Nanoceria Supported Palladium(0) Nanoparticles: Superb Catalyst in Dehydrogenation of Formic Acid at Room. Temperature. Appl. Catal. B.

[15] Gu XJ, Lu ZH, Jiang HL, Akita T, Xu Q (2011). Synergistic Catalysis of Metal-Organic Framework-Immobilized Au-Pd Nanoparticles in Dehydrogenation of Formic Acid for Chemical Hydrogen Storage. J. Am. Chem. Soc..

[16] Caner N (2017). Atomic Layer Deposition-SiO_2_ Layers Protected PdCoNi Nanoparticles Supported on TiO_2_ Nanopowders: Exceptionally Stable Nanocatalyst for the Dehydrogenation of Formic Acid. Appl. Catal. B.

[17] Gunasekar GH, Park K, Jung K-D, Yoon S (2016). Recent Developments in the Catalytic Hydrogenation of CO_2_ To Formic Acid/Formate Using Heterogeneous Catalysts. Inorg. Chem. Front..

[18] Song FZ, Zhu QL, Tsumori N, Xu Q (2015). Diamine-Alkalized Reduced Graphene Oxide: Immobilization of Sub-2 nm Palladium Nanoparticles and Optimization of Catalytic Activity for Dehydrogenation of Formic Acid. ACS Catal..

[19] Wiener H, Blum J, Feilchenfeld H, Sasson Y, Zalmanov N (1988). The Heterogeneous Catalytic-Hydrogenation Of Bicarbonate To Formate In Aqueous-Solutions. J. Catal..

[20] Mori K, Dojo M, Yamashita H (2013). Pd and Pd-Ag Nanoparticles within a Macroreticular Basic Resin: An Efficient Catalyst for Hydrogen Production from Formic Acid Decomposition. ACS Catal..

[21] Liu PL (2017). Visible-light-driven Catalytic Activity Enhancement of Pd in AuPd Nanoparticles for Hydrogen Evolution From Formic Acid at Room Temperature. Appl. Catal. B.

[22] Mori K (2017). Phenylamine-Functionalized Mesoporous Silica Supported Pdag Nanoparticles: A Dual Heterogeneous Catalyst For Formic Acid/CO_2_-Mediated Chemical Hydrogen Delivery/Storage. Chem. Commun..

[23] Masuda S, Mori K, Futamura Y, Yamashita H (2018). PdAg Nanoparticles Supported on Functionalized Mesoporous Carbon: Promotional Effect of Surface Amine Groups in Reversible Hydrogen Delivery/Storage Mediated by Formic Acid/CO2. ACS Catal..

[24] Mori K, Tanaka H, Dojo M, Yoshizawa K, Yamashita H (2015). Synergic Catalysis of PdCu Alloy Nanoparticles within a Macroreticular Basic Resin for Hydrogen Production from Formic Acid. Chem. Eur. J..

[25] Wang ZL (2013). An Efficient CoAuPd/C Catalyst for Hydrogen Generation from Formic Acid at Room Temperature. Angew. Chem. Int. Ed..

[26] Kohsuke Mori KN, Masuda S, Miyawaki K (2017). Hiromi Yamashita. Palladium Copper Chromium Ternary Nanoparticles Constructed *In Situ* Within A Basic Resin: Enhanced Activity In The Dehydrogenation Of Formic Acid. ChemCatChem.

[27] Mori K, Sano T, Kobayashi H, Yamashita H (2018). Surface Engineering of a Supported PdAg Catalyst for Hydrogenation of CO_2_ to Formic Acid: Elucidating the Active Pd Atoms in Alloy Nanoparticles. J. Am. Chem. Soc..

[28] Liu HL (2016). Controllable Encapsulation of “Clean” Metal Clusters within MOFs through Kinetic Modulation: Towards Advanced Heterogeneous Nanocatalysts. Angew. Chem. Int. Ed..

[29] Zhang N, Xu YJ (2013). Aggregation- and Leaching-Resistant, Reusable, and Multifunctional Pd@CeO_2_ as a Robust Nanocatalyst Achieved by a Hollow Core-Shell Strategy. Chem. Mater..

[30] Yang QH, Xu Q, Jiang HL (2017). Metal-Organic Frameworks Meet Metal Nanoparticles: Synergistic Effect For Enhanced Catalysis. Chem. Soc. Rev..

[31] Wen MC, Mori K, Kuwahara Y, An TC, Yamashita H (2017). Design and Architecture of Metal Organic Frameworks for Visible Light Enhanced Hydrogen Production. Appl. Catal. B.

[32] Esken D, Turner S, Lebedev OI, Van Tendeloo G, Fischer RA (2010). Au@ZIFs: Stabilization and Encapsulation of Cavity-Size Matching Gold Clusters inside Functionalized Zeolite Imidazolate Frameworks, ZIFs. Chem. Mater..

[33] Zhang WN (2014). A Family of Metal-Organic Frameworks Exhibiting Size-Selective Catalysis with Encapsulated Noble-Metal Nanoparticles. Adv. Mater..

[34] Huang YB (2014). Bimetallic Alloy Nanocrystals Encapsulated In Zif-8 For Synergistic Catalysis Of Ethylene Oxidative Degradation. Chem. Commun..

[35] Li S (2016). Template-Directed Synthesis of Porous and Protective Core–Shell Bionanoparticles. Angew. Chem. Int. Ed..

[36] Dai HM (2015). Synergistic Catalysis of AgPd@ZIF-8 on Dehydrogenation of Formic Acid. Appl. Catal. B.

[37] Wen MC (2015). Synthesis Of Ce Ions Doped Metal-Organic Framework For Promoting Catalytic H_2_ Production From Ammonia Borane Under Visible Light Irradiation. J. Mater. Chem. A.

[38] Enomoto T, Ueno S, Hosono E, Hagiwara M, Fujihara S (2017). Size-Controlled Synthesis Of Zif-8 Particles And Their Pyrolytic Conversion Into Zno Aggregates As Photoanode Materials Of Dye-Sensitized Solar Cells. Cryst. Eng. Commun..

[39] White RJ, Luque R, Budarin VL, Clark JH, Macquarrie DJ (2009). Supported Metal Nanoparticles on Porous Materials. Methods and Applications. Chem. Soc. Rev..

[40] Filonenko GA, Vrijburg WL, Hensen EJM, Pidko EA (2016). On the Activity ff Supported Au Catalysts in the Liquid Phase Hydrogenation of CO_2_ to Formates. J. Catal..

[41] Wen M, Mori K, Kuwahara Y, Yamashita H (2017). Plasmonic Au@Pd Nanoparticles Supported on a Basic Metal-Organic Framework: Synergic Boosting of H-2 Production from Formic Acid. Acs Energy Lett..

[42] Nguyen LTM (2015). Catalytic CO_2_ Hydrogenation To Formic Acid Over Carbon Nanotube-Graphene Supported Pdni Alloy Catalysts. Rsc Adv..

[43] Cheng J (2017). Achieving Efficient Room-temperature Catalytic H_2_ Evolution from Formic Acid through Atomically Controlling the Chemical Environment of Bimetallic Nanoparticles Immobilized by Isoreticular Amine-functionalized Metal-organic Frameworks. Appl. Catal. B.

[44] Loges B, Boddien A, Gartner F, Junge H, Beller M (2010). Catalytic Generation of Hydrogen from Formic acid and its Derivatives: Useful Hydrogen Storage Materials. Top. Catal..

[45] Martis M, Mori K, Fujiwara K, Ahn WS, Yamashita H (2013). Amine-Functionalized MIL-125 with Imbedded Palladium Nanoparticles as an Efficient Catalyst for Dehydrogenation of Formic Acid at Ambient Temperature. J. Phys. Chem. C.

[46] Navlani-Garcia M, Mori K, Wen MC, Kuwahara Y, Yamashita H (2015). Size Effect of Carbon-Supported Pd Nanoparticles in the Hydrogen Production from Formic Acid. Bull. Chem. Soc. Jpn.

[47] Delley B (1990). An All - Electron Numerical Method For Solving The Local Density Functional For Polyatomic Molecules. J. Chem. Phys..

[48] Delley B (2000). From Molecules To Solids With The Dmol^3^ Approach. J. Chem. Phys..

