# Supplementary material for: PdAg Nanoparticles within Core-Shell Structured Zeolitic Imidazolate Framework as a Dual Catalyst for Formic Acid-based Hydrogen Storage/Production
Source: Sci Rep. 2019 Oct 30;9:15675. doi: 10.1038/s41598-019-52133-5 (PMC6821696; doi:10.1038/s41598-019-52133-5)
Supplement: Supplementary file 1 — Supporing Inforamtion [file 41598_2019_52133_MOESM1_ESM.docx]

**PdAg Nanoparticles within Core-Shell Structured Zeolitic Imidazolate Framework as a Dual Catalyst for Formic Acid-based Hydrogen Storage/Production**

Meicheng Wen,^1,4^ Kohsuke Mori,^1,2,3^* Yuya Futamura,^1^ Yasutaka Kuwahara,^1,3^ Miriam Navlani-García,^1^ Taicheng An,^4^ and Hiromi Yamashita^1,3^*

^1^Division of Materials and Manufacturing Science, Graduate School of Engineering, Osaka University, 2-1 Yamadaoka, Suita, Osaka 565-0871, Japan

^2^JST, PRESTO, 4-1-8 Honcho, Kawaguchi, Saitama, 332-0012, Japan

^3^Elements Strategy Initiative for Catalysts Batteries (ESICB), Kyoto University, Katsura, Kyoto 615-8520, Japan

^4^Guangzhou Key Laboratory of Environmental Catalysis and Pollution Control, School of Environmental Science and Engineering Institute of Environmental Health and Pollution control, Guangdong University of Technology, Guangdong, 51006, China.

Corresponding Author

* Kohsuke Mori

mori@mat.eng.osaka-u.ac.jp

***Hiromi Yamashita**

**yamashita@mat.eng.osaka-u.ac.jp**


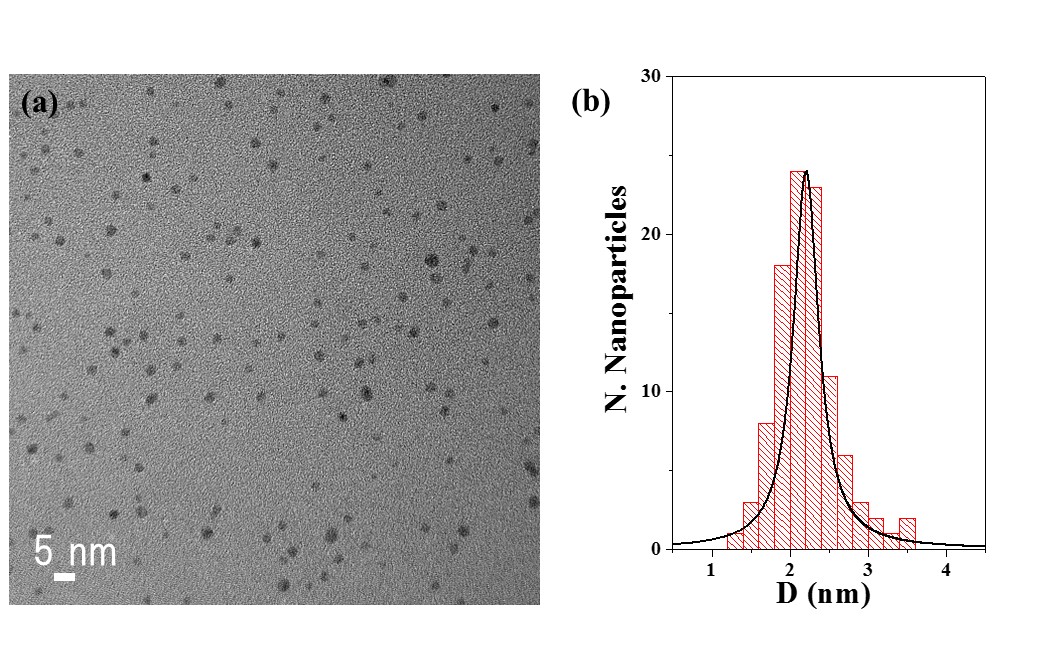
**Figure S1**. (A) TEM image and (B) size distribution diagram of PdAg alloy nanoparticles

**(B)**

**(A)**

**Table S1**. Curve Fitting Results for Pd and Ag K-Edge EXAFS Data

| sample | edge | shell | *CN* | *CN_total_* | *R*/Å | Δσ^2^/Å^2^ |
| --- | --- | --- | --- | --- | --- | --- |
| ZIF-8@Pd_1_Ag_2_@ZIF-8 | Pd K | Pd–Pd | 3.3 | 7.7 | 2.77 | 0.075 |
|  |  | Pd–Ag | 4.4 |  | 2.80 | 0.087 |
|  | Ag K | Ag–Ag | 5.1 | 9.6 | 2.84 | 0.084 |
|  |  | Ag–Pd | 4.5 |  | 2.80 | 0.020 |


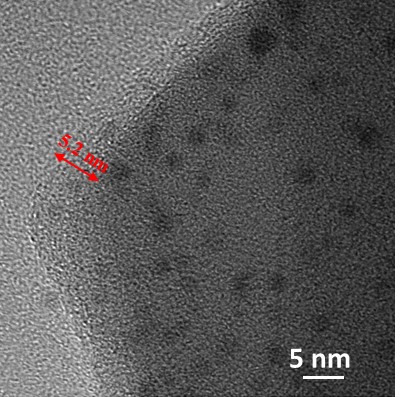


**Figure S2**. TEM image of Pd_1_Ag_2_@ZIF-8.

**Table S2**. The lattice spacing of (111) and (20-1) plane of different samples.

| Sample | (111) | (20-1) |
| --- | --- | --- |
| Ag | 2.36 Å | 1.83 Å |
| Pd | 2.25 Å | 1.74 Å |
| PdAg | 2.31 Å | 1.77 Å |


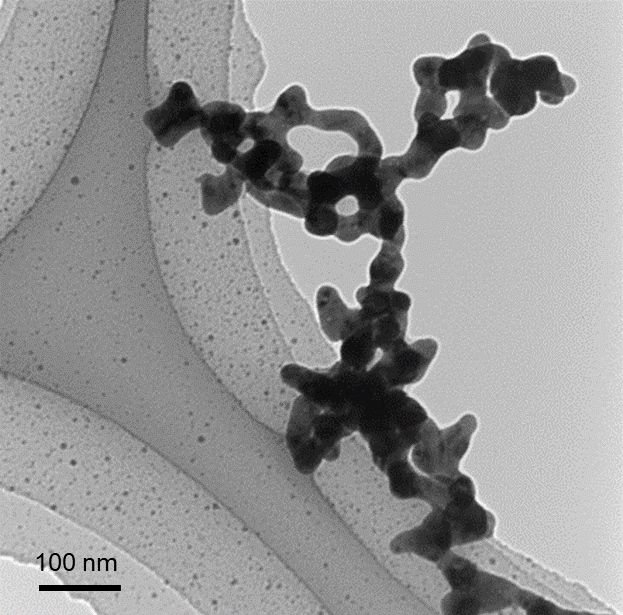


**Figure S3**. TEM image of colloidal Pd_1_Ag_2_ nanoparticles after reaction of the CO_2_ hydrogenation into FA.

**Figure S4**. TEM image of PdAg/ZIF-8-1 (a) and PdAg/ZIF-8-2 (c) and the size distribution of PdAg/ZIF-8-1 (b) and PdAg/ZIF-8-2 (d).


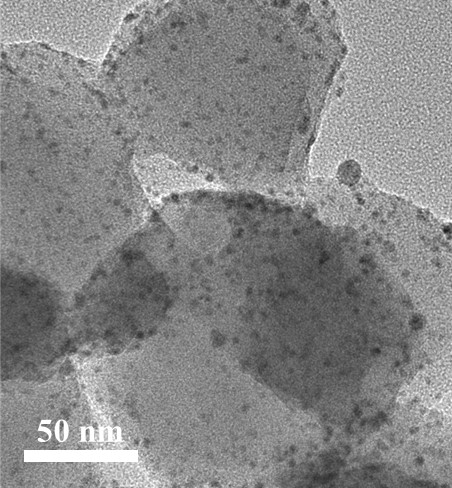


**Figure S5**. TEM image of ZIF-8@Pd_1_Ag_2_@ZIF-8 after the CO_2_ hydrogenation into FA.

**Figure S6**. The stability test of ZIF-8@Pd_1_Ag_2_@ZIF-8.

**Table S3**. Kinetic isotope effect in the dehydrogenation of FA.

| Catalyst | FA | Reaction rate (ummol∙min^-1^) | *k*_H_/*k*_D_ |
| --- | --- | --- | --- |
| Pd@ZIF-8 | HCOOH | 3.64 |  |
|  | HCOOD | 2.79 | 1.30 |
|  | DCOOH | 1.07 | 3.40 |
| ZIF-8@Pd_1_Ag_2_@ZIF-8 | HCOOH | 3.85 |  |
|  | HCOOD | 3.08 | 1.25 |
|  | DCOOH | 1.42 | 2.71 |


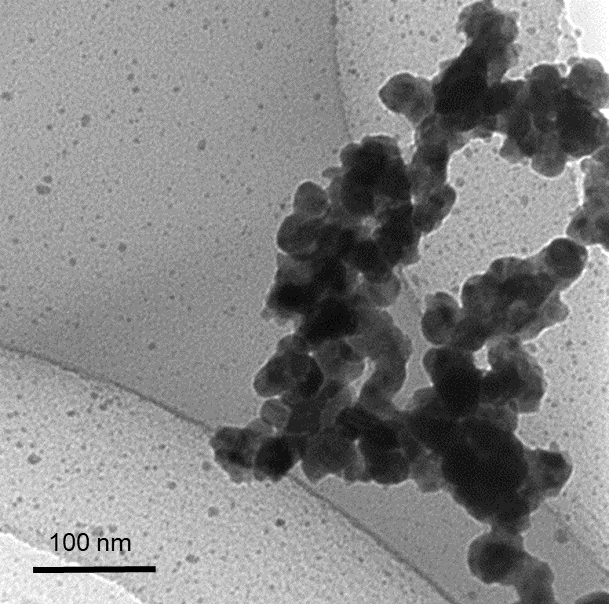


**Figure S7**. TEM image of colloidal Pd_1_Ag_2_ nanoparticles after reaction of FA decomposition.
